# Lactate-to-albumin ratio is associated with in-hospital mortality in patients with spontaneous subarachnoid hemorrhage and a nomogram model construction

**DOI:** 10.3389/fneur.2022.1009253

**Published:** 2022-10-17

**Authors:** Guo-Guo Zhang, Jia-Hui Hao, Qi Yong, Qian-Qian Nie, Gui-Qiang Yuan, Zong-Qing Zheng, Jin-Quan Li

**Affiliations:** ^1^Department of Neurosurgery, The First Affiliated Hospital of Soochow University, Suzhou, China; ^2^Department of Internal Medicine, The Seventh Affiliated Hospital of University of South of China, Changsha, China; ^3^Department of Neurology, The First Affiliated Hospital of Soochow University, Suzhou, China; ^4^Department of Neurosurgery, Changshu No.2 People's Hospital, Changshu, China

**Keywords:** spontaneous subarachnoid hemorrhage, lactate, albumin, nomogram, in-hospital mortality

## Abstract

**Introduction:**

Subarachnoid hemorrhage (SAH) is a severe hemorrhagic stroke with high mortality. However, there is a lack of clinical tools for predicting in-hospital mortality in clinical practice. LAR is a novel clinical marker that has demonstrated prognostic significance in a variety of diseases.

**Methods:**

Critically ill patients diagnosed and SAH with their data in the Medical Information Mart for Intensive Care-IV (MIMIC-IV) database and the eICU Collaborative Research Database (eICU-CRD) were included in our study. Multivariate logistic regression was utilized to establish the nomogram.

**Results:**

A total of 244 patients with spontaneous SAH in the MIMIC-IV database were eligible for the study as a training set, and 83 patients in eICU-CRD were included for external validation. Data on clinical characteristics, laboratory parameters and outcomes were collected. Univariate and multivariate logistic regression analysis identified age (OR: 1.042, *P*-value: 0.003), LAR (OR: 2.592, *P*-value: 0.011), anion gap (OR: 1.134, *P*-value: 0.036) and APSIII (OR: 1.028, *P*-value: < 0.001) as independent predictors of in-hospital mortality and we developed a nomogram model based on these factors. The nomogram model incorporated with LAR, APSIII, age and anion gap demonstrated great discrimination and clinical utility both in the training set (accuracy: 77.5%, AUC: 0.811) and validation set (accuracy: 75.9%, AUC: 0.822).

**Conclusion:**

LAR is closely associated with increased in-hospital mortality of patients with spontaneous SAH, which could serve as a novel clinical marker. The nomogram model combined with LAR, APSIII, age, and anion gap presents good predictive performance and clinical practicability.

## Introduction

Subarachnoid hemorrhage (SAH) is a severe subtype of hemorrhagic stroke, accounting for 5% of patients with stroke (1). The rupture of an intracranial aneurysm is the primary cause in the majority of cases, SAH in one-third of patients can be fatal, and at least 20% of survivors do not regain functional independence (2). Most survivors after SAH suffer from long-term physical disabilities and psychological disorders (3, 4). Despite extensive clinical studies and maximal treatment, patients with SAH still present with a dismal clinical outcome (5, 6). Due to the high mortality and low recovery rates, it is critical to establish a useful nomogram for predicting prognosis in patients with SAH.

Currently, over 40 classical grading score systems have been developed and widely used to assess the severity level after SAH, including Glasgow Coma Scale (GCS), the Hunt–Hess scale (HH), the World Federation of Neurosurgical Societies (WFNS) scale, the modified Fisher Scale (mFS), and Subarachnoid Hemorrhage Early Brain Edema Score (SEBES) (7–14). These grading score systems are concise, but the differences between the grades for each system are ambiguous and subjective. Moreover, most of them do not include personal objective indicators, like age, vital signs, and laboratory findings, which may play an independent prognostic role in SAH. In addition, none of these clinical scales can be applied to directly predict mortality in in-hospital patients with SAH. Thus, there is a need to construct a novel grading score system that includes personal-related and prognostic parameters to directly predict the clinical outcome of in-hospital patients with SAH, which would contribute to the clinical decision in an early phase of SAH treatment.

In clinical practice, the serum lactate level is commonly used to evaluate tissue hypoxia and is presented as a key indicator for organ failure and mortality of patients with several critical illnesses, including sepsis, trauma, and pediatric critical illness (15–18). Patients after SAH suffer from low peripheral oxygenation, leading to anaerobic glycolysis due to insufficient oxygen delivery, which leads to lactate production (19). In addition, albumin is mainly synthesized in liver cells and plays a significant role in serum substantial transportation and maintaining the balance of plasma colloid osmotic pressure (20). Patients with SAH frequently become hypovolemic and hemodynamically impaired a few days after symptom onset. Administration of serum albumin after SAH contributes to improving clinical outcomes (21). Importantly, there is emerging evidence that a novel marker termed “LAR,” that is, the ratio of lactate to albumin, is established and preferably used in predicting mortality of critically ill patients (22), including cardiac arrest (23), shock, sepsis (24), and traumatic brain injury (25). However, the prognostic value of LAR in patients with spontaneous SAH remains unclear.

Therefore, this study aimed to explore the association between LAR and the prognosis of patients with SAH and evaluate the predictive utility of LAR combined with the objective hematological indicators. In addition, a nomogram based on LAR was established to improve the discriminating ability of the well-known prognostic parameters in SAH.

## Materials and methods

### Data source

We extracted patient data from the Medical Information Mart for Intensive Care (MIMIC-IV) database (26) and the eICU Collaborative Research Database (eICU-CRD) (27). MIMIC-IV is a publicly available database comprising electronic health records of 53,423 patients in critical care units from the Beth Israel Deaconess Medical Center (BIDMC, Boston, MA) between 2008 and 2019. The eICU database is a multi-center ICU database containing structured EHR data of over 200,000 admissions in 2014 and 2015 in 208 U.S. hospitals. One of the authors (GZ) has obtained access to both the databases and was responsible for data extraction (Certification number: 45824512). This study was conducted according to the REporting of studies Conducted using Observational Routinely-collected health Data (RECORD) statement (28).

### Participant selection

We restricted the search of patients with primary diagnoses of spontaneous SAH using the International Classification of Diseases (ICD-9 code =“430” and ICD-10 code =“I60”). People younger than 18 years, without ICU admission, and with missing baseline data, which accounted for more than 20%, were excluded from the study. For patients with multiple ICU and hospital admissions, we only included data from the first ICU admission of the first hospitalization.

### Data extraction and preprocessing

All data in this study were obtained from the database using structured query language (SQL) by PostgreSQL (version 9.4.6). The data extraction included demographic parameters (age and gender), vital signs (temperature, heart rate, respiratory rate, and systolic blood pressure), laboratory parameters (blood routine examination, serum biochemicals, coagulation function, plasmic electrolytes, and arterial blood gas analysis), comorbidity (myocardial infarction, congestive heart failure, chronic pulmonary disease, diabetes, liver disease, and renal disease), Acute Physiology Score III (APSIII), and survival information. In the data preprocessing step, all variables were collected within 24 h of ICU admission, missing values were first filled with first measurements during this hospitalization. For other missing data, categorical variables are filled with mode, and continuous variables are filled with median.

### Statistical analysis

Statistical analysis was performed using SPSS 23.0 and STATA 17.0. Baseline characteristics of all patients were divided by survival state. Continuous variables are presented as mean ± standard deviation (if normal) or medians with interquartile ranges (if non-normal), and categorical variables are presented as percentage. Differences between two groups were compared using the *t*-test or Wilcoxon rank sum test for continuous variables, and the chi-square test for categorical variables, and variables with *p*-values < 0.05 were included in the binary logistic regression model. Univariable (non-adjusted) and multivariable (adjusted) binary logistic regression models were used to assess the association between clinical characteristics and prognosis of patients. Variables with *p*-values < 0.05 in the multivariate logistic regression model were used to construct the final prediction model. A nomogram was then constructed based on the logistic prediction model. Receiver operating characteristic (ROC) curve analysis was used to assess the prediction model performance in the training (MIMIC-IV) and testing (eICU) cohorts. The area under the ROC curve (AUC) was used to summarize diagnostic accuracy (AUC = 0.5 no discrimination and AUC = 1 perfect discrimination), and the accuracy, sensitivity, specificity, negative predictive value, and positive predictive value were also calculated. In addition, decision curve analysis (DCA) was applied to evaluate the clinical usefulness of the models.

## Results

### Baseline characteristics

**Figure 1:**
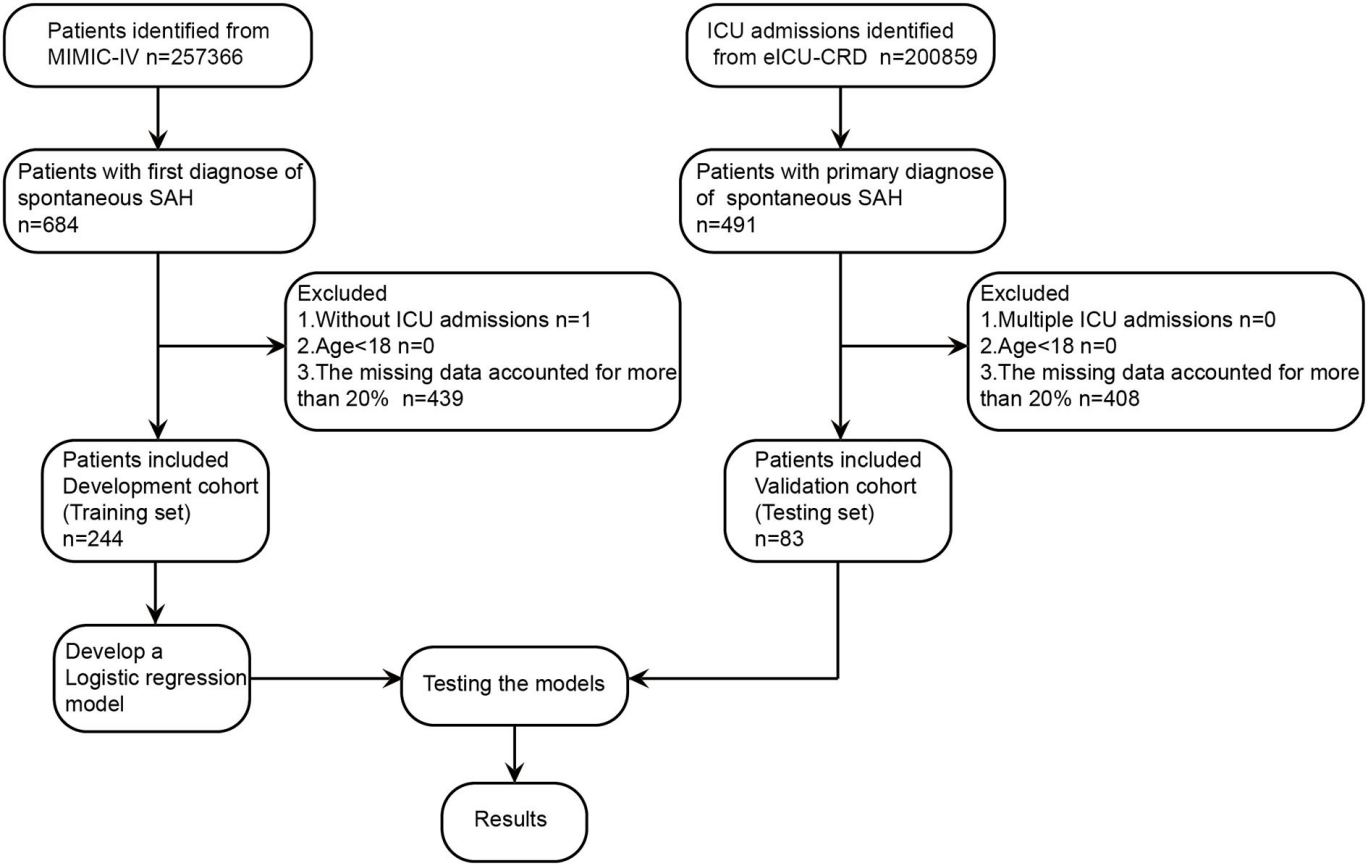
Study design and study flowchart. eICU-CRD, eICU Collaborative Research Database; ICU, intensive care unit; MIMIC-IV, Medical Information Mart for Intensive Care-IV; SAH, subarachnoid hemorrhage.

As shown in Figure 1, a total of 244 patients with spontaneous SAH in with data present in MIMIC-IV were enrolled in this study according to the aforementioned inclusion and exclusion criteria. These patients were divided into two groups according to the in-hospital survival state (174 patients were included in the survivor group, and 70 patients in the non-survivor group), and the baseline characteristics are listed in Table 1. An independent cohort of 83 patients with spontaneous SAH in the eICU-CRD was included for external validation (Supplementary Table S2). Compared with the survivor group, the non-survivor group was more likely to be elderly and have higher respiratory rate (RR), systolic blood pressure (SBP), aspartate aminotransferase (AST), blood urea nitrogen (BUN), glucose, lactate-to-albumin ratio (LAR), anion gap, and Acute Physiology Score III (APSIII), whereas they tended to have lower base excess (BE) and bicarbonate. These results suggest that these clinical characteristics may be related to the prognosis of patients.

**Table 1:** Demographic and baseline characteristics of study patients.

**Characteristics**	**Overall (*n* = 244)**	**Alive (*n* = 174)**	**Death (*n* = 70)**	***P*–value**
Age (years)	59.3 ± 13.7	57.4 ± 13.2	64.1 ± 13.8	<0.001
Sex				0.960
Female	147 (60.2%)	105 (60.3%)	42 (60%)	
Male	97 (39.8%)	69 (29.7%)	28 (40%)	
Vital signs				
Temperature (°C)	37.2 (36.1–37.9)	37.2 (36.2–37.9)	36.9 (35.8–38.0)	0.149
Heart rate (bpm)	99(76–112)	98(73–111)	104(89–119)	0.081
Respiratory rate (bpm)	26(18–31)	25(18–29)	27(22–34)	0.042
SBP (mmHg)	129 ± 23.9	131 ± 23.2	123 ± 24.9	0.010
Laboratory findings				
RBC (10^9^ /L)	4.05 ± 0.66	4.04 ± 0.58	4.09 ± 0.83	0.613
Hemoglobin (g/dl)	12.2 ± 1.84	12.2 ± 1.72	12.2 ± 2.12	0.973
WBC (10^9^ /L)	12.7 (9.73–15.9)	12.6(9.78–15.1)	13.1(9.68–18.1)	0.116
Platelet (10^9^ /L)	233 (187–290)	236 (191–288)	222 (173–303)	0.553
ALT (U/L)	28.0 (18.0–48.5)	28.0(18.0–42.5)	28.0(16.0–55.0)	0.658
AST (U/L)	33.0 (24.0–49.8)	33.0 (23.0–43.3)	41.0 (26.0–66.5)	0.018
BUN (mg/dL)	14.0 (10.0–18.0)	13.0(10.0–18.0)	15.0(11.0–19.3)	0.025
Cr (mg/dL)	0.80(0.60–1.00)	0.80(0.60–0.90)	0.80(0.70–1.10)	0.125
Glucose (mmol/L)	142 (121–174)	138(117–165)	155(129–200)	0.004
Lactate (mmol/L)	2.00(1.33–3.00)	1.90(1.20–2.53)	2.50(1.70–3.95)	<0.001
Albumin (g/dl)	3.63 ± 0.61	3.75 ± 0.54	3.33 ± 0.67	<0.001
LAR	0.56(0.38–0.79)	0.50(0.33–0.70)	0.74(0.55–1.10)	<0.001
Sodium (mmol/L)	140 (137–143)	140 (137–142)	140 (138–143)	0.303
Chloride (mmol/L)	106 (103–109)	106 (102–109)	107 (104–111)	0.053
Potassium (mmol/L)	3.70(3.50–3.90)	3.70(3.50–3.90)	3.70(3.38–4.00)	0.693
Calcium (mmol/L)	1.10(1.05–1.20)	1.11(1.05–1.18)	1.10(1.02–1.20)	0.306
PT (second)	12.5(11.7–13.4)	12.5(11.7–13.4)	12.6(11.6–13.7)	0.503
APTT (second)	27.4(24.9–30.9)	27.4(25.0–30.8)	27.5(24.7–32.6)	0.593
INR	1.10(1.10–1.20)	1.10(1.00–1.20)	1.10(1.10–1.23)	0.233
PaO2 (mmHg)	155(95.3–212)	155(86.8–203)	160(107–239)	0.143
PaCO2 (mmHg)	38.0(34.0–42.0)	38.0(34.0–42.0)	39.0(32.0–43.3)	0.673
Bicarbonate (mmol/L)	23.0(21.0–25.0)	23.0(21.0–25.0)	22.0(20.0–24.0)	0.028
BE (mEq/L)	0.00(−2.00–0.00)	0.00(−2.00–0.00)	−1.00(−5.00–0.00)	0.018
Anion gap (mmol/L)	14.0(13.0–16.0)	14.00(12.8–16.0)	15.5(13.0–18.0)	0.005
Coexisting disorders				
Myocardial infarction	18 (7.4%)	12 (6.9%)	6 (8.6%)	0.651
Congestive heart failure	14 (5.7%)	10 (5.7%)	4 (5.7%)	0.992
Chronic pulmonary disease	43 (17.6%)	28 (16.1%)	15 (21.4%)	0.322
Diabetes	39 (16%)	30 (17.2%)	9 (12.9%)	0.398
Liver disease	11 (4.5%)	5 (2.9%)	6 (8.6%)	0.052
Renal disease	14 (5.7%)	8 (4.6%)	6 (8.6%)	0.227
APSIII score	46.0(30.0–68.8)	38.50(27.0–60.0)	61.50(44.0–86.5)	<0.001

SBP, Systolic Blood Pressure; RBC, Red blood cell; WBC, White blood cell; ALT, Alanine transaminase; AST, Aspartate aminotransferase; BUN, Blood urea nitrogen; Cr, Creatinine; LAR, lactate albumin ratio; PT, Prothrombin time; APTT, Activated partial thromboplastin time; INR, International normalized ratio; BE, Base excess; APSIII score, Acute Physiology III score.

### Association between clinical characteristics and hospital mortality

Then, we used both univariate and multivariate logistic regression analyses to evaluate the association between these clinical characteristics and hospital mortality. As shown in Table 2, a total of nine variables (age, respiratory rate, SBP, BUN, glucose, LAR, bicarbonate, anion gap, and APSIII) were statistically significant (*p*-value < 0.05) in the univariate logistic regression analysis. Then, the variables were included in the multivariate logistic regression analysis for further analysis. In the multivariate logistic regression analysis, the result revealed that age (adjusted OR, 95% CI, *p*-value: 1.042, 1.014–1.070, 0.003), LAR (adjusted OR, 95% CI, *p*-value: 2.592, 1.243–5.406, 0.011), anion gap (adjusted OR, 95% CI, *p*-value: 1.134, 1.008–1.276, 0.036), and APSIII (adjusted OR, 95% CI, *p*-value: 1.028, 1.013–1.043, < 0.001) were independent prognostic factors of hospital mortality in patients with spontaneous SAH after adjustment. These variables were then included in the construction of the new prediction model.

**Table 2:** Univariate and multivariate logistic regression analysis of factors associated with SAH.

	**Univariate analysis**		**Multivariate analysis**	
	**OR (95% Cl)**	***P*-value**	**OR (95% Cl)**	***P*-value**
Age	1.039(1.016–1.063)	0.001	1.042(1.014–1.070)	0.003
Respiratory rate	1.030(1.001–1.060)	0.045	1.006(0.972–1.042)	0.724
SBP	0.984(0.972–0.996)	0.012	0.987(0.973–1.002)	0.082
AST	1.003(0.999–1.006)	0.117	1.001(0.998–1.004)	0.512
BUN	1.040(1.009–1.071)	0.010	1.004(0.965–1.044)	0.850
Glucose	1.005(1.001–1.009)	0.009	1.001(0.996–1.006)	0.689
LAR	4.206(2.208–8.013)	<0.001	2.592(1.243–5.406)	0.011
Bicarbonate	0.917(0.847–0.993)	0.032	1.062(0.939–1.201)	0.339
BE	0.959(0.898–1.025)	0.221	0.999(0.925–1.079)	0.981
Anion gap	1.146(1.053–1.247)	0.002	1.134(1.008–1.276)	0.036
APSIII score	1.038(1.025–1.052)	<0.001	1.028(1.013–1.043)	<0.001

SBP, Systolic Blood Pressure; AST, Aspartate aminotransferase; BUN, Blood urea nitrogen; LAR, lactate albumin ratio; BE, Base excess; APSIII score, Acute Physiology III score; OR, odds ratio; CI, confidence interval.

### Nomogram for predicting in-hospital mortality risk

**Figure 2:**
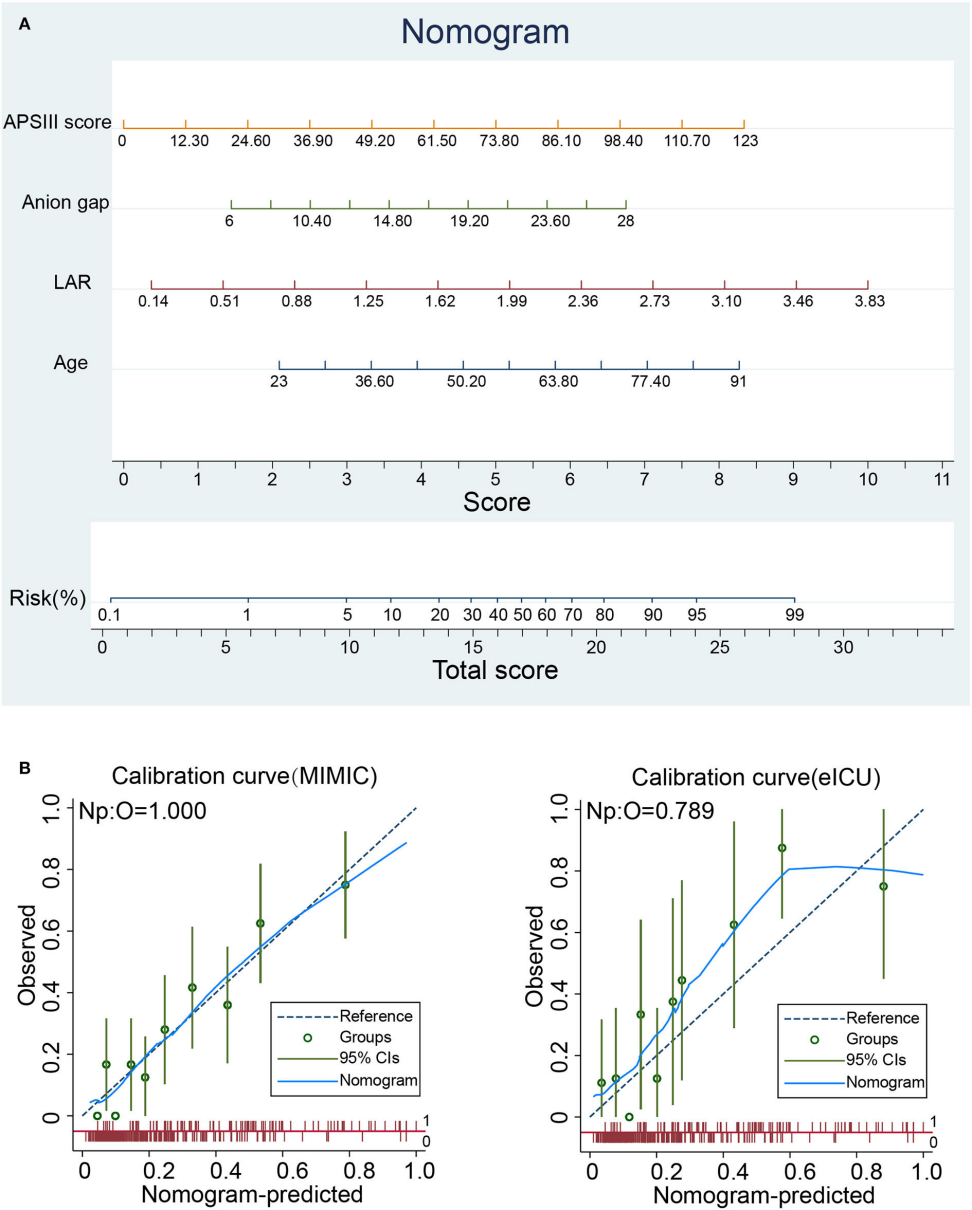
Nomogram analyses of patients with spontaneous SAH. **(A)** Nomogram for predicting in-hospital mortality of patients with spontaneous SAH. **(B)** Calibration curve of the nomogram predicting in-hospital mortality in the training and validation cohorts. APSIII, Acute Physiology Score III; LAR, lactate-to-albumin ratio.

Based on the results of the multivariate logistic regression analysis, a predictive nomogram (Figure 2A) was constructed to evaluate the risk of in-hospital death. The following four variables were incorporated to develop the prognostic nomogram: age, LAR, anion gap, and APSIII. Each patient will get a total point plus the point of five prognostic variables based on this nomogram, which corresponds to the prediction of the risk of in-hospital death. To compare the consistency between the probability of in-hospital mortality predicted by the nomogram model and actual outcomes, calibration curves were plotted. As shown in Figure 2B, the calibration curves of the nomogram were close to the standard curves in the training cohort and validation cohort, which suggested that the nomogram had great consistency.

### Predictive performance and clinical usefulness evaluation of the nomogram

**Figure 3:**
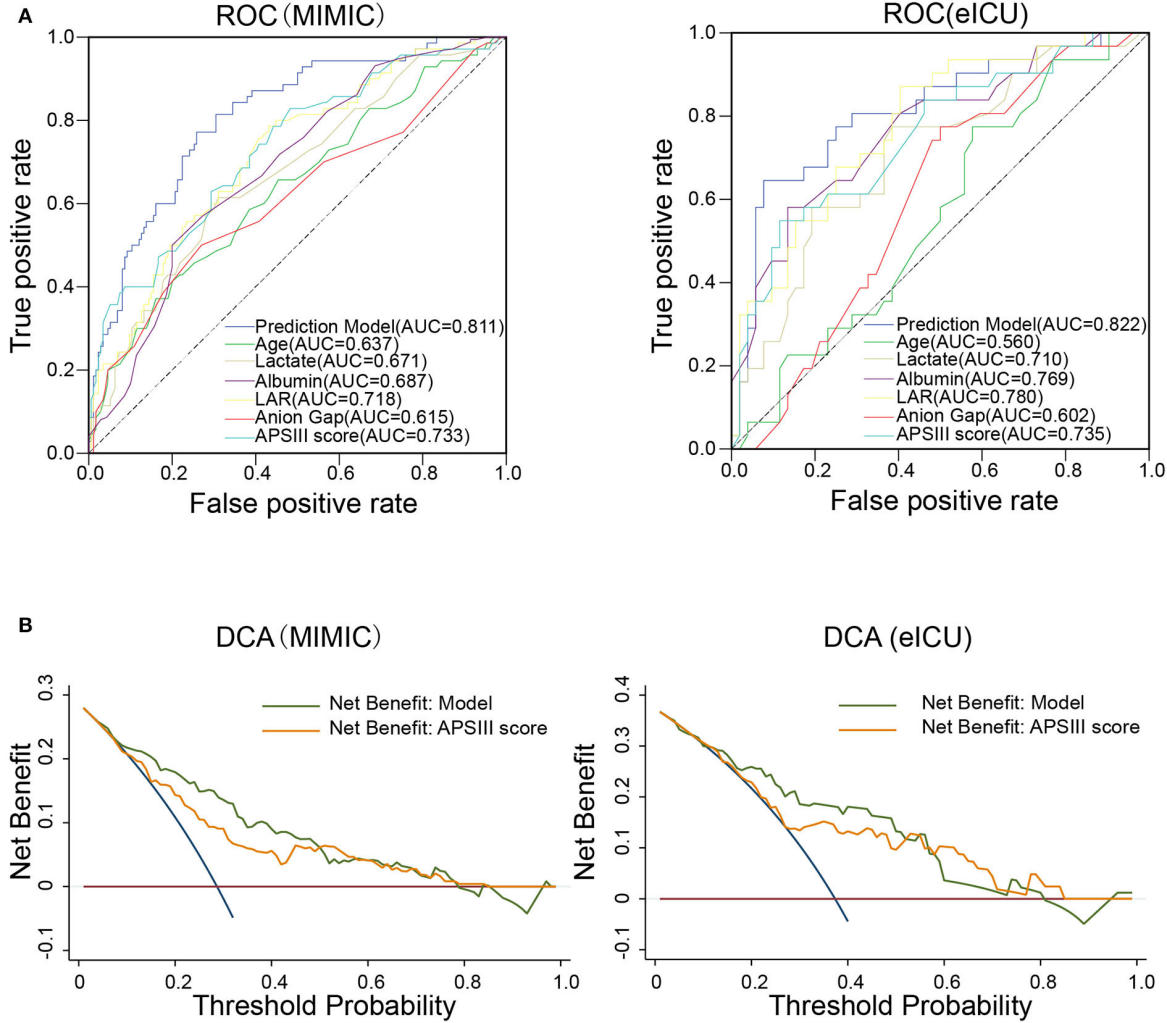
Predictive performance of the prediction model. **(A)** Receiver operating characteristic (ROC) curve of different prognostic variables compared with the prediction model for the predictive performance of the in-hospital mortality in the training set (MIMIC-IV database) and validation set (eICU database). **(B)** Decision curve analysis (DCA) of the prediction model compared with APSIII in the training set and validation set.

To compare the predictive effect of different indexes, receiver operating characteristic (ROC) curve analysis was performed to quantify the performance of different indexes in the training set (MIMIC-IV) and validation set (eICU). As shown in Figure 3A and Table 3, the AUC value of LAR in the training group was 0.718. The accuracy, sensitivity, specificity, PPV, and NPV of the prediction model were 0.750, 0.215, 0.966, 0.714, and 0.753, respectively. The AUC value of APSIII in the training group was 0.733. The accuracy, sensitivity, specificity, PPV, and NPV of APSIII were 0.775, 0.357, 0.943, 0.714, and 0.785, respectively. The AUC value of the prediction model in the training group was 0.811. The accuracy, sensitivity, specificity, PPV, and NPV of the prediction model were 0.775, 0.414, 0.920, 0.674, and 0.796, respectively. Then, the predictive accuracy of the prediction model was further evaluated in a validation set. The AUC, accuracy, sensitivity, specificity, PPV, and NPV of LAR were 0.780, 0.723, 0.290, 0.981, 0.900, and 0.699, respectively. The AUC, accuracy, sensitivity, specificity, PPV, and NPV of APSIII were 0.735, 0.723, 0.452, 0.885, 0.700, and 0.730, respectively. The AUC, accuracy, sensitivity, specificity, PPV, and NPV of the prediction model were 0.822, 0.759, 0.452, 0.942, 0.824, and 0.742, respectively. These results show that LAR had good predictive power in patients with severe SAH and that the new model combining with age, anion gap, LAR, and APSIII had a better predictive power than individual APSIII.

**Table 3:** Comparisons of different predictive index.

**Dataset**	**Model**	**AUC**	**Accuracy**	**Sensitivity**	**Specificity**	**PPV**	**NPV**
MIMIC–IV	Model	0.811(0.752–0.869)	0.775(0.718–0.823)	0.414(0.306–0.531)	0.920(0.869–0.951)	0.674(0.525–0.795)	0.796(0.735–0.846)
	Age	0.637(0.558–0.715)	0.721(0.662–0.774)	0.043(0.015–0.119)	0.994(0.968–0.999)	0.750(0.301–0.954)	0.721(0.661–0.774)
	Lactate	0.671(0.596–0.746)	0.721(0.662–0.774)	0.100(0.049–0.192)	0.971(0.935–0.988)	0.583(0.320–0.807)	0.728(0.668–0.782)
	Albumin	0.687(0.611–0.763)	0.750(0.692–0.800)	0.257(0.169–0.370)	0.948(0.905–0.973)	0.667(0.478–0.814)	0.760(0.699–0.812)
	LAR	0.718(0.648–0.788)	0.750(0.692–0.800)	0.215(0.134–0.324)	0.966(0.927–0.984)	0.714(0.501–0.862)	0.753(0.693–0.805)
	Anion Gap	0.615(0.533–0.698)	0.730(0.671–0.781)	0.100(0.049–0.192)	0.983(0.951–0.994)	0.700(0.397–0.892)	0.731(0.671–0.784)
	APSIII score	0.733(0.663–0.803)	0.775(0.718–0.823)	0.357(0.255–0.474)	0.943(0.897–0.969)	0.714(0.550–0.837)	0.785(0.724–0.835)
eICU	Model	0.822(0.726–0.918)	0.759(0.657–0.838)	0.452(0.292–0.622)	0.942(0.844–0.980)	0.824(0.590–0.938)	0.742(0.626–0.833)
	Age	0.560(0.436–0.685)	0.627(0.519–0.723)	0.065(0.018–0.207)	0.962(0.870–0.989)	0.500(0.150–0.850)	0.633(0.523–0.731)
	Lactate	0.710(0.594–0.825)	0.675(0.568–0.766)	0.161(0.071–0.326)	0.981(0.899–0.997)	0.833(0.437–0.970)	0.662(0.551–0.758)
	Albumin	0.769(0.663–0.875)	0.711(0.606–0.797)	0.645(0.470–0.789)	0.750(0.618–0.848)	0.606(0.437–0.753)	0.780(0.648–0.873)
	LAR	0.780(0.679–0.881)	0.723(0.618–0.808)	0.290(0.161–0.466)	0.981(0.899–0.997)	0.900(0.596–0.982)	0.699(0.586–0.792)
	Anion Gap	0.602(0.481–0.724)	0.602(0.495–0.701)	0.000(0.000–0.1103)	0.962(0.870–0.989)	0.000(0.000–0.658)	0.617(0.508–0.716)
	APSIII score	0.735(0.620–0.849)	0.723(0.618–0.808)	0.452(0.292–0.622)	0.885(0770–0.946)	0.700(0.481–0.855)	0.730(0.610–0.824)

LAR, lactate albumin ratio; APSIII score, Acute Physiology III score; AUC, Area under the curve; PPV, Positive predictive value; NPV, Negative predictive value.

We further constructed a DCA curve to predict the reliability of the nomogram. The DCA curve (Figure 3B) showed that the nomogram provides the higher clinical benefit in predicting in-hospital mortality than APSIII in both the training and validation sets.

## Discussion

Recently, LAR, calculated by the ratio of lactate to albumin in human serum, is widely used as a prognostic indicator in patients with various critical illnesses, such as cardiac arrest, sepsis, and trauma (23–25). Patients after SAH occurrence undergo life-threatening processes in hospital, which warrants a need to develop an early and prognostic nomogram for predicting the clinical outcome of in-hospital patients with SAH. In this study, we investigated the clinical application of the lactate/albumin (L/A) ratio to be as an early prognostic marker for in-hospital patients with SAH from two large cohorts in two open-source databases. Our results demonstrated that a higher LAR is correlated with poor clinical outcomes in patients with SAH. The multivariate regression analysis indicated that LAR is an independent risk factor for patients with SAH after adjusting for other confounding factors. A novel nomogram established based on LAR presented a higher predictive value than the APSIII scoring system, which is commonly used for patients with SAH.

Lactate is a product of anaerobic respiration, which is produced in large amounts under hypoxia, and is positively correlated with mortality (29–31). Patients after SAH occurrence commonly experience sympathetic hyperactivation, which may result in metabolism dysfunction, especially hyperlactatemia (32). Extensive data had proved that blood lactate concentrations have prognostic implications in patients with SAH (33–36), which is similar to our result that serum lactate presented an independent predictive factor for in-hospital patients with SAH. However, as systemic lactate is influenced by many other factors, the prognostic role of lactate in SAH still remains controversial. Some studies have showed that lactate is related to the severity of SAH in patients with SAH but did not show an association with prognosis (37). Thus, the clinical value of lactate alone in SAH still remains controversial.

Moreover, SAH occurrence is usually accompanied with neurological and systemic inflammation in patients with SAH (38, 39). Most patients with SAH undergo the unfavorable outcome due to the brain injury that caused by subarachnoid blood and subsequent hemoglobin degradation induced inflammatory cascade (38). Albumin contributes to the generation of anti-inflammatory substances such as lipoxins, resolvins, and protectins, which can accelerate the wound healing process and inhibit disease progression (40), providing a reasonable explanation for the protective effect of albumin in patients with SAH. In this study, we have also included albumin as a parameter in our model. The results indicated that albumin plays a beneficial role in the outcome of patients with SAH, which is in accordance with a previous study in which the serum albumin level presented a predictive role in neurologic deficits and clinical outcomes for patients with spontaneous SAH (41).

More importantly, as lactate and albumin are produced from different organs and influenced by multiple mechanisms, a combined index of LAR can reduce the impact of a single factor on the regulation mechanism (22). To the best of our knowledge, this is the first study to explore the prognostic value of LAR among in-hospital patients with SAH. Our results confirmed that the predictive value of LAR can be better than lactate or albumin alone in outcomes of in-hospital patients with SAH. Currently, for in-hospital patients with early-phase SAH, the Acute Physiology Score III (APSIII) including the classical GCS score system is derived from acute physiology and chronic health evaluation scoring system III (APACHE III), which is commonly used for SAH disease severity assessment (42). In this study, we found that LAR alone presented a prognostic value similar to APSIII in the prediction of SAH clinical outcomes. In addition, based on the multivariate analysis, we constructed a novel predictive nomogram that includes independent factors like age, anion gap, LAR, and APSIII. Our data demonstrated that the new predictive model that combined LAR and other independent factors showed a superior performance compared with the APSIII score system alone in assessing the outcome of in-hospital SAH, suggesting a beneficial clinical value of this nomogram.

There are some limitations in our study. On the one hand, hospital mortality was included as one of the prognostic indexes, and long-term clinical outcome and patients' survival status were not recorded due to the limitations of these open-source medical databases. A further study with a detailed long-term survival time of patients with SAH should be performed to determine the predictive value of the LAR prognostic model. On the other hand, the drugs and hospital medical care, which may have an impact on the level of LAR in patients with SAH, were not recorded, which might exert a confounding effect on our conclusion.

## Conclusions

LAR may serve as a critical parameter for the prognosis of patients with spontaneous SAH. The nomogram prediction model combined with LAR, APSIII, and other prognostic factors can predict in-hospital mortality of patients with SAH with high degrees of accuracy The application of the predictive model could help clinicians judge the patients' condition, thereby guiding treatment.

## Data availability statement

The data analyzed in this study was obtained from the Medical Information Mart for Intensive Care IV (MIMIC-IV) database, the following licenses/restrictions apply: To access the files, you must be a credentialed user, complete the required training (CITI Data or Specimens Only Research) and sign the data use agreement for the project. Requests to access these datasets should be directed to PhysioNet, https://doi.org/10.13026/7vcr-e114.

## Author contributions

J-QL and Z-QZ: conceptualization (lead) and supervision (lead). G-GZ and J-HH: conceptualization (equal), data curation (lead), formal analysis (lead), writing—original draft (lead), and writing—review and editing (equal). QY, Q-QN, and G-QY: conceptualization (supporting), methodology (lead), and writing—review and editing (equal). All authors contributed to the article and approved the submitted version.

## Conflict of interest

The authors declare that the research was conducted in the absence of any commercial or financial relationships that could be construed as a potential conflict of interest.

## Publisher's note

All claims expressed in this article are solely those of the authors and do not necessarily represent those of their affiliated organizations, or those of the publisher, the editors and the reviewers. Any product that may be evaluated in this article, or claim that may be made by its manufacturer, is not guaranteed or endorsed by the publisher.

## References

[1] Macdonald RL Schweizer TA. Spontaneous subarachnoid haemorrhage. Lancet (London, England). (2017) 389:655–66. 10.1016/S0140-6736(16)30668-7 27637674

[2] Al-Khindi T Macdonald RL Schweizer TA. Cognitive and functional outcome after aneurysmal subarachnoid hemorrhage. Stroke. (2010) 41:e519–36. 10.1161/STROKEAHA.110.581975 20595669

[3] Taufique Z May T Meyers E Falo C Mayer SA Agarwal S. Predictors of poor quality of life 1 year after subarachnoid hemorrhage. Neurosurgery. (2016) 78:256–64. 10.1227/NEU.0000000000001042 26421590

[4] Lawton MT Vates GE. Subarachnoid hemorrhage. N Engl J Med. (2017) 377:257–66. 10.1056/NEJMcp1605827 28723321

[5] Maher M Schweizer TA Macdonald RL. Treatment of spontaneous subarachnoid hemorrhage: guidelines and gaps. Stroke. (2020) 51:1326–32. 10.1161/STROKEAHA.119.025997 31964292

[6] Suzuki H Nakano F. To improve translational research in subarachnoid hemorrhage. Transl Stroke Res. (2018) 9:1–3. 10.1007/s12975-017-0546-2 28620886

[7] Rosen DS Macdonald RL. Subarachnoid hemorrhage grading scales: a systematic review. Neurocrit Care. (2005) 2:110–8. 10.1385/NCC:2:2:110 16159052

[8] de Oliveira Manoel AL Jaja BN Germans MR Yan H Qian W Kouzmina E. The VASOGRADE: a simple grading scale for prediction of delayed cerebral ischemia after subarachnoid hemorrhage. Stroke. (2015) 46:1826–31. 10.1161/STROKEAHA.115.008728 25977276

[9] Mourelo-Fariña M Pértega S Galeiras R A. Model for prediction of in-hospital mortality in patients with subarachnoid hemorrhage. Neurocrit Care. (2021) 34:508–18. 10.1007/s12028-020-01041-y 32671649

[10] Lee VH Ouyang B John S Conners JJ Garg R Bleck TP. Risk stratification for the in-hospital mortality in subarachnoid hemorrhage: the HAIR score. Neurocrit Care. (2014) 21:14–9. 10.1007/s12028-013-9952-9 24420695

[11] Ahn SH Savarraj JP Pervez M Jones W Park J Jeon SB. The Subarachnoid Hemorrhage Early Brain Edema Score Predicts Delayed Cerebral Ischemia and Clinical Outcomes. Neurosurgery. (2018) 83:137–45. 10.1093/neuros/nyx364 28973675

[12] Frontera JA Claassen J Schmidt JM Wartenberg KE Temes R Connolly ES Jr., et al. Prediction of symptomatic vasospasm after subarachnoid hemorrhage: the modified fisher scale. Neurosurgery. (2006). 59:21–7. 10.1227/01.neu.0000243277.86222.6c 16823296

[13] Oshiro EM Walter KA Piantadosi S Witham TF Tamargo RJ. A new subarachnoid hemorrhage grading system based on the Glasgow Coma Scale: a comparison with the Hunt and Hess and World Federation of Neurological Surgeons Scales in a clinical series. Neurosurgery. (1997) 41:140–7. 10.1097/00006123-199707000-00029 9218306

[14] Hunt WE Hess RM. Surgical risk as related to time of intervention in the repair of intracranial aneurysms. J Neurosurg. (1968) 28:14–20. 10.3171/jns.1968.28.1.0014 5635959

[15] Thanachartwet V Desakorn V Sahassananda D Jittmittraphap A Oer-Areemitr N Osothsomboon S. Serum procalcitonin and peripheral venous lactate for predicting dengue shock and/or organ failure: a prospective observational study. PLoS Negl Trop Dis. (2016) 10:e0004961. 10.1371/journal.pntd.0004961 27564863 PMC5001649

[16] Sobhian B Kröpfl A Hölzenbein T Khadem A Redl H Bahrami S. Increased circulating D-lactate levels predict risk of mortality after hemorrhage and surgical trauma in baboons. Shock (Augusta, Ga). (2012) 37:473–7. 10.1097/SHK.0b013e318249cb96 22266971

[17] Mikkelsen ME Miltiades AN Gaieski DF Goyal M Fuchs BD Shah CV. Serum lactate is associated with mortality in severe sepsis independent of organ failure and shock. Crit Care Med. (2009) 37:1670–7. 10.1097/CCM.0b013e31819fcf68 19325467

[18] Bai Z Zhu X Li M Hua J Li Y Pan J. Effectiveness of predicting in-hospital mortality in critically ill children by assessing blood lactate levels at admission. BMC Pediatr. (2014) 14:83. 10.1186/1471-2431-14-83 24673817 PMC3976355

[19] Macdonald RL. Delayed neurological deterioration after subarachnoid haemorrhage. Nature Rev Neurol. (2014) 10:44–58. 10.1038/nrneurol.2013.246 24323051

[20] De Simone G di Masi A Ascenzi P. Serum albumin: a multifaced enzyme. Int J Molecular Sci. (2021) 22:1810086. 10.3390/ijms221810086 34576249 PMC8466385

[21] Suarez JI Shannon L Zaidat OO Suri MF Singh G Lynch G. Effect of human albumin administration on clinical outcome and hospital cost in patients with subarachnoid hemorrhage. J Neurosurg. (2004) 100:585–90. 10.3171/jns.2004.100.4.0585 15070109

[22] Gharipour A Razavi R Gharipour M Mukasa D. Lactate/albumin ratio: An early prognostic marker in critically ill patients. Am J Emerg Med. (2020) 38:2088–95. 10.1016/j.ajem.2020.06.067 33152585

[23] Kong T Chung SP Lee HS Kim S Lee J Hwang SO. The prognostic usefulness of the lactate/albumin ratio for predicting clinical outcomes in out-of-hospital cardiac arrest: a prospective, multicenter observational study (koCARC) study. Shock (Augusta, Ga). (2020) 53:442–51. 10.1097/SHK.0000000000001405 31306348

[24] Bou Chebl R Jamali S Sabra M Safa R Berbari I Shami A. Lactate/Albumin ratio as a predictor of in-hospital mortality in septic patients presenting to the emergency department. Front Med. (2020) 7:550182. 10.3389/fmed.2020.550182 33072780 PMC7536276

[25] Wang R He M Qu F Zhang J Xu J. Lactate albumin ratio is associated with mortality in patients with moderate to severe traumatic brain injury. Front Neurol. (2022) 13:662385. 10.3389/fneur.2022.662385 35432157 PMC9011050

[26] Goldberger AL Amaral LA Glass L Hausdorff JM Ivanov PC Mark RG. PhysioBank, physiotoolkit, and physionet: components of a new research resource for complex physiologic signals. Circulation. (2000) 101:E215–E20. 10.1161/01.CIR.101.23.e215 10851218

[27] Pollard TJ Johnson AEW Raffa JD Celi LA Mark RG Badawi O. The eICU collaborative research database, a freely available multi-center database for critical care research. Sci Data. (2018) 5:180178. 10.1038/sdata.2018.178 30204154 PMC6132188

[28] Benchimol EI Smeeth L Guttmann A Harron K Moher D Petersen I. The reporting of studies conducted using observational routinely-collected health data (RECORD) statement. PLoS Med. (2015) 12:e1001885. 10.1371/journal.pmed.1001885 26440803 PMC4595218

[29] Fuller BM Dellinger RP. Lactate as a hemodynamic marker in the critically ill. Curr Opin Crit Care. (2012) 18:267–72. 10.1097/MCC.0b013e3283532b8a 22517402 PMC3608508

[30] Leverve XM. Energy metabolism in critically ill patients: lactate is a major oxidizable substrate. Curr Opin Clin Nutr Metab Care. (1999) 2:165–9. 10.1097/00075197-199903000-00013 10453349

[31] Kruse O Grunnet N Barfod C. Blood lactate as a predictor for in-hospital mortality in patients admitted acutely to hospital: a systematic review. Scand J Trauma Resusc Emerg Med. (2011) 19:74. 10.1186/1757-7241-19-74 22202128 PMC3292838

[32] Bakker J Nijsten MW Jansen TC. Clinical use of lactate monitoring in critically ill patients. Ann Intensive Care. (2013) 3:12. 10.1186/2110-5820-3-12 23663301 PMC3654944

[33] Ndieugnou Djangang N Ramunno P Izzi A Garufi A Menozzi M Diaferia D. The prognostic role of lactate concentrations after aneurysmal subarachnoid hemorrhage. Brain Sci. (2020) 10:121004. 10.3390/brainsci10121004 33348866 PMC7766816

[34] van Donkelaar CE Dijkland SA van den Bergh WM Bakker J Dippel DW Nijsten MW. Early circulating lactate and glucose levels after aneurysmal subarachnoid hemorrhage correlate with poor outcome and delayed cerebral ischemia: a two-center cohort study. Crit Care Med. (2016) 44:966–72. 10.1097/CCM.0000000000001569 26751612

[35] Aisiku IP Chen PR Truong H Monsivais DR Edlow J. Admission serum lactate predicts mortality in aneurysmal subarachnoid hemorrhage. Am J Emerg Med. (2016) 34:708–12. 10.1016/j.ajem.2015.12.079 26818152

[36] Oh CH Kim JW Kim GH Lee KR Hong DY Park SO. Serum lactate could predict mortality in patients with spontaneous subarachnoid hemorrhage in the emergency department. Front Neurol. (2020) 11:975. 10.3389/fneur.2020.00975 33013645 PMC7499023

[37] Poblete RA Cen SY Zheng L Emanuel BA. Serum lactic acid following aneurysmal subarachnoid hemorrhage is a marker of disease severity but is not associated with hospital outcomes. Front Neurol. (2018) 9:593. 10.3389/fneur.2018.00593 30083130 PMC6064931

[38] de Oliveira Manoel AL Macdonald RL. Neuroinflammation as a target for intervention in subarachnoid hemorrhage. Front Neurol. (2018) 9:292. 10.3389/fneur.2018.00292 29770118 PMC5941982

[39] Tam AK Ilodigwe D Mocco J Mayer S Kassell N Ruefenacht D. Impact of systemic inflammatory response syndrome on vasospasm, cerebral infarction, and outcome after subarachnoid hemorrhage: exploratory analysis of CONSCIOUS-1 database. Neurocrit Care. (2010) 13:182–9. 10.1007/s12028-010-9402-x 20593247

[40] Das UN. Albumin and lipid enriched albumin for the critically ill. J Assoc Physicians India. (2009) 57:53–9. 19753760

[41] Kapoor A Dhandapani S Gaudihalli S Dhandapani M Singh H. Mukherjee KK. Serum albumin level in spontaneous subarachnoid hemorrhage: more than a mere nutritional marker! *Br J Neurosurg*. (2018) 32:47–52. 10.1080/02688697.2017.1344615 28658989

[42] Navarrete-Navarro P Rivera-Fernández R López-Mutuberría MT Galindo I Murillo F Dominguez JM. Outcome prediction in terms of functional disability and mortality at 1 year among ICU-admitted severe stroke patients: a prospective epidemiological study in the south of the European Union (Evascan Project, Andalusia, Spain). Intensive Care Med. (2003) 29:1237–44. 10.1007/s00134-003-1755-6 12756437

