# Supplementary material for: Lactate-to-albumin ratio is associated with in-hospital mortality in patients with spontaneous subarachnoid hemorrhage and a nomogram model construction
Source: Front Neurol. 2022 Oct 17;13:1009253. doi: 10.3389/fneur.2022.1009253 (PMC9618723; doi:10.3389/fneur.2022.1009253)
Supplement: Supplementary file 1 [file Data_Sheet_1.doc]

**Supplementary table 1 The baseline characteristics of the included and excluded patients.**

| Characteristics | Included (n=244) | | Excluded (n=440) | | P-value |
| --- | --- | --- | --- | --- | --- |
|  | Value | Lost count | Value | Lost count |  |
| Age (years) | 59.3±13.7 | 0 | 59.5±14.9 | 0 | 0.908 |
| Sex |  |  |  |  | 0.560 |
| Female | 147(60.2%) | 0 | 255(58.0%) | 0 |  |
| Male | 97(39.8%) | 0 | 185(42.0%) | 0 |  |
| Vital signs |  |  |  |  |  |
| Temperature (℃) | 37.2(36.1-37.9) | 3 | 37.1(36.3-37.5) | 7 | 0.271 |
| Heart rate (bpm) | 99(76-112) | 0 | 99(77-109) | 0 | 0.619 |
| Respiratory rate (bpm) | 26(18-31) | 0 | 27(15-31) | 0 | 0.126 |
| SBP (mmHg) | 129±23.9 | 0 | 130±21.2 | 0 | 0.405 |
| Laboratory findings |  |  |  |  |  |
| RBC (10^9^ /L) | 4.06±0.66 | 1 | 4.10±0.57 | 34 | 0.350 |
| Hemoglobin (g/dl) | 12.2±1.84 | 1 | 12.5±1.67 | 34 | 0.076 |
| WBC (10^9^ /L) | 12.7 (9.70-15.9) | 1 | 12.6(10.3-15.1) | 34 | 0.565 |
| Platelet (10^9^ /L) | 233(187-290) | 1 | 235(201-282) | 36 | 0.362 |
| ALT (U/L) | 28.0 (16.3-52.0) | 16 | 28.0(21.0-48.5) | 311 | 0.106 |
| AST (U/L) | 32.5 (23.0-57.0) | 14 | 34.0 (28.0-46.0) | 310 | 0.192 |
| BUN (mg/dL) | 14.0 (10.0-18.0) | 1 | 14.0(11.0-17.0) | 33 | 0.598 |
| Cr (mg/dL) | 0.80(0.60-1.00) | 1 | 0.80(0.70-0.90) | 33 | 0.101 |
| Glucose (mmol/L) | 142 (120-176) | 6 | 141(121-166) | 63 | 0.560 |
| Lactate (mmol/L) | 2.00(1.30-3.10) | 6 | 1.98(1.58-2.68) | 159 | 0.279 |
| Albumin (g/dl) | 3.63±0.61 | 0 | 3.64±0.48 | 350 | 0.861 |
| LAR | 0.56(0.35-0.85) | 6 | 0.57(0.44-1.01) | 429 | 0.365 |
| Sodium (mmol/L) | 140(137-143) | 1 | 140(138-142) | 32 | 0.572 |
| Chloride (mmol/L) | 106(103-109) | 1 | 106(103-109) | 32 | 0.462 |
| Potassium (mmol/L) | 3.70(3.30-4.00) | 16 | 3.80(3.40-4.10) | 291 | 0.184 |
| Calcium (mmol/L) | 1.09(1.03-1.15) | 12 | 1.09(1.04-1.14) | 278 | 0.935 |
| PT (second) | 12.5(11.7-13.4) | 4 | 12.5(11.9-13.4) | 63 | 0.312 |
| APTT (second) | 27.4(24.8-31.0) | 5 | 27.8(25.5-30.1) | 65 | 0.786 |
| INR | 1.10(1.03-1.20) | 4 | 1.10(1.00-1.30) | 62 | 0.093 |
| PaO2 (mmHg) | 155(92.0-216) | 12 | 166(99.0-256) | 257 | 0.069 |
| PaCO2 (mmHg) | 38.0(34.0-43.0) | 12 | 38.0(33.0-43.0) | 257 | 0.438 |
| Bicarbonate (mmol/L) | 23.0(21.0-25.0) | 1 | 23.0(21.0-25.0) | 33 | 0.266 |
| BE (mEq/L) | 0.00(-3.00-0.00) | 11 | 0.00(-2.00-1.00) | 257 | 0.089 |
| Anion gap (mmol/L) | 14.0(13.0-16.0) | 0 | 14.00(12.0-16.0) | 33 | 0.917 |
| Coexisting disorders |  |  |  |  |  |
| Myocardial infarction | 18 (7.4%) | 0 | 32 (7.3%) | 0 | 0.960 |
| Congestive heart failure | 14 (5.7%) | 0 | 22 (5.0%) | 0 | 0.679 |
| Chronic pulmonary disease | 43 (17.6%) | 0 | 74 (16.8%) | 0 | 0.789 |
| Diabetes | 39 (16%) | 0 | 68 (15.5%) | 0 | 0.461 |
| Liver disease | 11 (4.5%) | 0 | 10 (2.3%) | 0 | 0.105 |
| Renal disease | 14 (5.7%) | 0 | 15 (3.4%) | 0 | 0.148 |
| APSIII score | 46.0(30.0-68.8) | 0 | 45.0(38.0-57.0) | 0 | 0.282 |

Note: SBP (Systolic Blood Pressure), RBC (Red blood cell), WBC (White blood cell), ALT (Alanine transaminase), AST (Aspartate aminotransferase), BUN (Blood urea nitrogen), Cr (Creatinine), LAR (lactate albumin ratio), PT (Prothrombin time), APTT (Activated partial thromboplastin time), INR (International normalized ratio), BE (Base excess), APSIII score (Acute Physiology III score)

**Supplementary table 2 Baseline characteristics of patients in the eICU cohorts**

| Characteristics | Overall  (N=83) | Alive  (n=52) | Death  (n=31) | P-value |
| --- | --- | --- | --- | --- |
| Age (years) | 57.70±15.66 | 56.27±16.58 | 60.10±13.90 | 0.284 |
| Sex |  |  |  |  |
| Female | 46(55.4%) | 27(51.9%) | 19(61.3%) | 0.406 |
| Male | 37(44.6%) | 25(48.1%) | 12(38.7%) |  |
| Heart rate (bpm) | 85.93±19.79 | 84.81±19.88 | 87.81±19.80 | 0.508 |
| Respiratory rate (bpm) | 18.00(17.00-22.00) | 18.00(16.25-23.00) | 19.00(17.00-22.00) | 0.770 |
| SBP (mmHg) | 130.00(117.00-153.00) | 131.50(117.75-145.75) | 127.00(106.00-154.00) | 0.785 |
| BUN (mg/dL) | 16.00(12.00-22.00) | 16.50(11.00-21.75) | 16.00(13.00-25.00) | 0.556 |
| Cr (mg/dL) | 0.80(0.65-1.12) | 0.70(0.60-1.08) | 0.99(0.76-1.16) | 0.016 |
| Glucose (mmol/L) | 146.00(124.00-199.00) | 138.00(113.25-190.25) | 181.00(136.00-226.00) | 0.013 |
| Lactate (mmol/L) | 1.56(1.10-2.90) | 1.40(0.90-2.10) | 2.50(1.50-3.80) | 0.001 |
| Albumin (g/dl) | 3.10(2.40-3.60) | 3.30(2.83-3.90) | 2.50(2.10-3.10) | <0.001 |
| LAR | 0.55(0.33-0.93) | 0.40(0.27-0.73) | 0.89(0.52-1.47) | <0.001 |
| Bicarbonate (mmol/L) | 23.42±3.39 | 23.88±3.37 | 22.65±3.33 | 0.109 |
| Anion gap (mmol/L) | 13.00(10.00-14.00) | 12.60(9.00-14.00) | 13.00(12.50-15.00) | 0.118 |
| APSIII score | 58.24±26.05 | 49.31±19.64 | 73.23±28.79 | <0.001 |

Note: SBP (Systolic Blood Pressure), BUN (Blood urea nitrogen), Cr (Creatinine), LAR (lactate albumin ratio), APSIII score (Acute Physiology III score)
